# User experience of a seizure risk forecasting app: A mixed methods investigation

**DOI:** 10.1016/j.yebeh.2024.109876

**Published:** 2024-06-07

**Authors:** Rachel E. Stirling, Ewan S. Nurse, Daniel Payne, Jodie Naim-Feil, Honor Coleman, Dean R. Freestone, Mark P. Richarson, Benjamin H. Brinkmann, Wendyl J. D’Souza, David B. Grayden, Mark J. Cook, Philippa J. Karoly

**Affiliations:** a Department of Biomedical Engineering, University of Melbourne, Melbourne, Victoria, Australia; b Graeme Clark Institute of Biomedical Engineering, University of Melbourne, Melbourne, Victoria, Australia; c Seer Medical, Melbourne, Victoria, Australia; d Department of Medicine, St Vincent’s Hospital Melbourne, The University of Melbourne, Australia; e Melbourne School of Psychological Sciences, The University of Melbourne, Melbourne, Victoria, Australia; f Epilepsy Research Centre, Department of Medicine (Austin Health), University of Melbourne, Victoria, Melbourne, Australia; g Department of Neuroscience, Faculty of Medicine, Nursing & Health Science, Monash University, Melbourne, Australia; h Division of Neuroscience, King’s College London, London, UK; i Department of Neurology, Mayo Foundation, Rochester, MN, United States

**Keywords:** Community survey, Patient experience, Mood questionnaires, Non-invasive wearables, Seizure forecasting devices

## Abstract

**Objective:**

Over recent years, there has been a growing interest in exploring the utility of seizure risk forecasting, particularly how it could improve quality of life for people living with epilepsy. This study reports on user experiences and perspectives of a seizure risk forecaster app, as well as the potential impact on mood and adjustment to epilepsy.

**Methods:**

Active app users were asked to complete a survey (baseline and 3-month follow-up) to assess perspectives on the forecast feature as well as mood and adjustment. Post-hoc, nine neutral forecast users (neither agreed nor disagreed it was useful) completed semi-structured interviews, to gain further insight into their perspectives of epilepsy management and seizure forecasting. Non-parametric statistical tests and inductive thematic analyses were used to analyse the quantitative and qualitative data, respectively.

**Results:**

Surveys were completed by 111 users. Responders consisted of “app users” (n = 58), and “app and forecast users” (n = 53). Of the “app and forecast users”, 40 % believed the forecast was accurate enough to be useful in monitoring for seizure risk, and 60 % adopted it for purposes like scheduling activities and helping mental state. Feeling more in control was the most common response to both high and low risk forecasted states. In-depth interviews revealed five broad themes, of which ‘frustrations with lack of direction’ (regarding their current epilepsy management approach), ‘benefits of increased self-knowledge’ and ‘current and anticipated usefulness of forecasting’ were the most common.

**Significance:**

Preliminary results suggest that seizure risk forecasting can be a useful tool for people with epilepsy to make lifestyle changes, such as scheduling daily events, and experience greater feelings of control. These improvements may be attributed, at least partly, to the improvements in self-knowledge experienced through forecast use.

## 1. Introduction

More than one-third of people with epilepsy continue to suffer uncontrolled seizures [1]. For these individuals, the fear of unpredictable seizures is a significant challenge that impinges on their autonomy, safety, and daily routines [2,3]. Consequently, seizure risk forecasting has emerged as a potential tool to help relieve the burden of unpredictable seizures on people with epilepsy, while also serving as a valuable tool for clinical decision-making. Seizure risk forecasting is the prediction of the risk of having a seizure within a certain timeframe (typically hours, days or weeks), which can be achieved by analysing seizure timing in conjunction with relevant physiological data such as long-term electroencephalography (EEG) [4–8] or heart rate [9,10].

While advances in technology (e.g., chronic recording technologies [5,11,12]) and data analysis methods (e.g., deep learning [13,14]) have greatly improved the field, the readiness of seizure risk forecasting for widespread use has not been fully established. Importantly, implementing seizure risk forecasting in a real-world scenario requires an understanding of user needs and perspectives and the real-life benefits of this technology. For example, it remains to be determined if seizure risk forecasting can assuage anxiety and fear without inadvertently encouraging risk-taking behaviours that could compromise safety. Greater understanding of this is imperative from both ethical and regulatory standpoints before widespread adoption of a forecasting system can occur.

To date, the vast majority of scientific understanding on patient perspectives and likely benefits of seizure risk forecasting systems derives from theoretical patient and caregiver surveys [2,15–20]. Combined, these conclude that the ability to forecast seizures is very important to people living with epilepsy [2,17,19], provided that it can reduce the impact of unpredictable seizures (e.g., improve confidence and independence) [17,19] and is aligned to the patient’s preference [2,15–20]. Theoretically, most people expect they will benefit from forecasted high risk and low risk periods, particularly for planning out one’s day [17].

While these theoretical perspectives are important, they may not mirror actual behaviour towards real-world forecasting systems [21]. Historically, only one clinical trial of a seizure prediction device has been conducted (NeuroVista seizure advisory system [5]). While this device instilled increased confidence and autonomy in most individuals assessed within the cohort (n = 4/6), one participant experienced distress and an unwelcome shift in their sense of identity [22]. Therefore, it is critical to investigate user experiences and perspectives of a forecasting system in larger cohorts, particularly patient-preferred non-invasive or minimally-invasive systems [16,19,20,23,24], as these may minimise potential feelings of distress and changes in identity. This and other critical challenges remain to be addressed for seizure forecasting to become the patient-centred clinical tool envisioned over the last few decades.

Recently, as part of the Epilepsy Foundation of America’s ‘My Seizure Gauge’ grant [25], a seizure risk forecast tool was piloted within a publicly accessible mobile epilepsy management app (Seer Medical Pty Ltd). The forecast feature provided a non-invasive assessment of seizure risk, based on historic cyclic patterns, which enabled users to visualise their future seizure likelihood over hours to days. This study aimed to explore user behaviour changes and perspectives of accuracy and risk state, in response to the seizure risk forecast tool, and to examine links between user experience/perspectives and mood and adjustment to epilepsy using a mix of quantitative and qualitative approaches.

## 2. Methodology

An observational cohort study was conducted with users of a mobile epilepsy management app. A mixed methods approach, involving quantitative (surveys) and qualitative (semi-structured interviews) methods, was used to assess user experiences and perspectives of the forecast feature within the mobile app.

Survey participants were recruited through convenience sampling by inviting “active app users” to complete a survey via Active app users were defined as users who had opened the mobile epilepsy management app in August 2022, but not necessarily with forecast feature access. Baseline responses were collected between September 2022 and October 2022. Three months after the final collection date, a follow-up survey was sent to all participants who completed the initial survey.

Additionally, semi-structured interviews were conducted with “neutral forecast users” who agreed to be interviewed. Neutral forecast users were defined as surveyed users who neither agreed nor disagreed that the forecast was accurate enough to be useful, based on their response to one of the survey questions. The purpose of the interviews was to gain a more detailed understanding of user’s experiences with and perspectives of epilepsy management. All interviewees had a confirmed epilepsy diagnosis and had access to the forecast feature in the epilepsy management app.

### 2.1. Participants

Surveys were emailed to 1207 active app users, with 115 responses. This response rate (9.5 %) is consistent with previous work [18,26]. Participants who did not complete all survey questions or were suspected duplicates (n = 4) were excluded from the analysis; all remaining responders were included in the analysis, leaving a total of 111 participants. At 3-month follow-up, the 111 participants were emailed a follow-up survey. Of these, 41 responded (37 %). Clinical and demographic information for responder and non-responder groups are reported in Table 1. Forecast performance comparisons between responder and non-responder groups are reported in Supplementary Appendix G.

All participants gave informed consent. Data were stored and processed on a secure cloud platform, compliant with management of protected health information. The study was approved by the St Vincent’s Hospital Melbourne Human Research Ethics Committee (HREC 165.19).

### 2.2. Materials

#### 2.2.1. Seizure risk forecasting tool

**Fig. 1.**
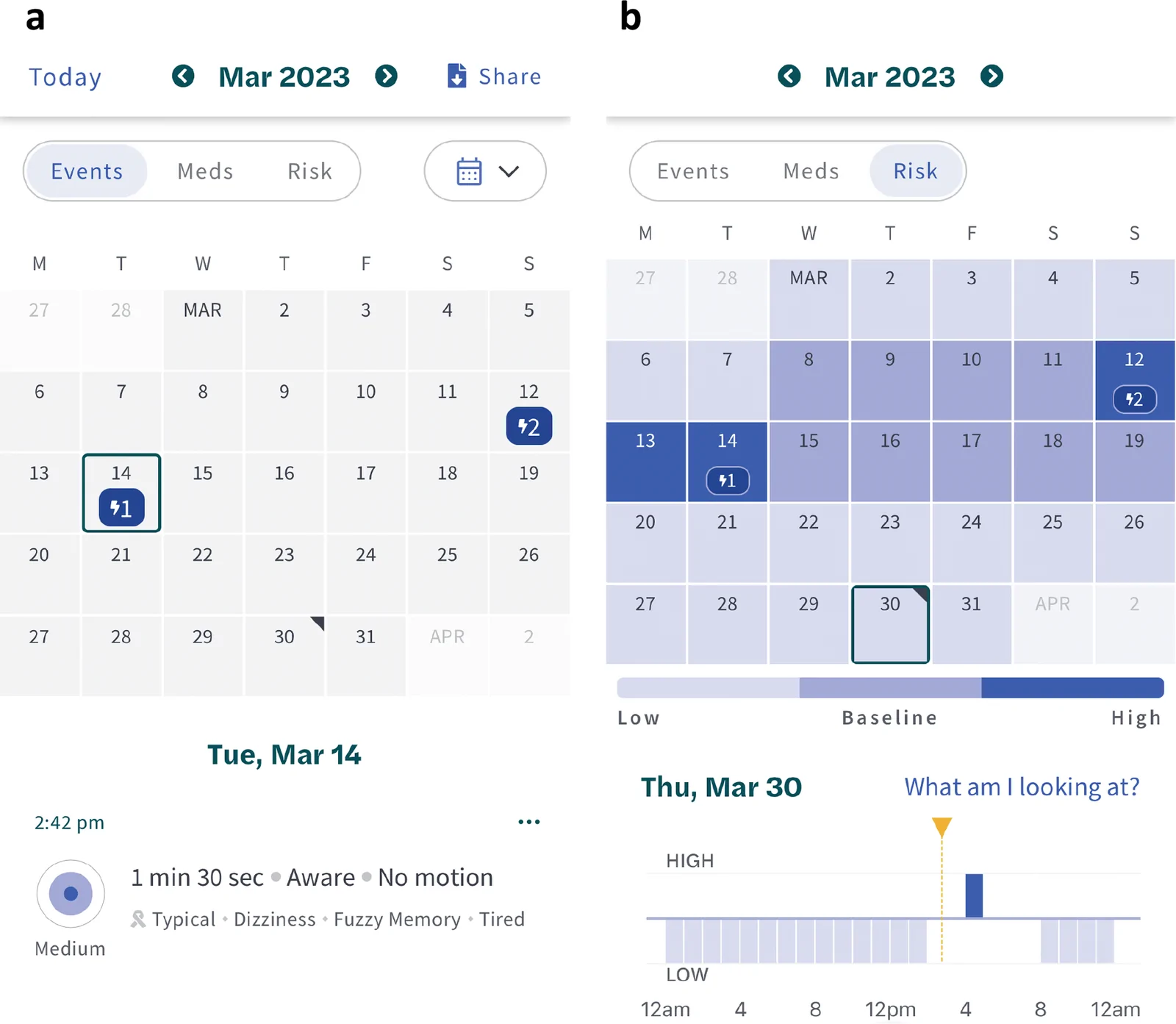
Seizure event diary (a) and seizure risk forecast feature (b) shown to users of a mobile app. **a:** Reported seizure events could be customised to include symptoms and characteristics, including seizure duration, clinical symptoms (awareness and motor activity), subjective symptoms (light-headedness, heart racing, tiredness, etc.) and perceived intensity. **b:** The seizure risk forecast incorporates a calendar view (shows daily risk levels) and a bar view (shows hourly risk levels), where darker colours indicate higher risk and lighter colours indicate lower risk.

A seizure risk forecasting feature was incorporated into an existing mobile epilepsy management app (Seer app, Seer Medical Pty Ltd) for app users that had reported at least 10 events (Fig. 1a) and achieved adequate cycle strength (n = 1480 users) between June 2022 and November 2023. The forecasting feature allowed users to observe their seizure risk on hourly (bar view) and daily (calendar view) timescales for 60 days into the future (Fig. 1b). The forecasting algorithm was based on a previously reported model [10], which used historic cyclic patterns in seizure timing and linked physiological data to produce hourly and daily forecasts. For details about the forecasting algorithm, see Supplementary Appendix A.

All app users were given clear information on the necessity of seeking advice from a medical professional regarding any clinical management decisions based on the seizure risk forecast.

#### 2.2.2. Survey

The survey comprised two sections: 1) seizure risk forecasting perspectives and 2) mood and adjustment questionnaires (Supplementary Fig. 1). The first question asked users how many times the forecast feature was viewed in the past month, which then divided responders into two main groups: “In Theory” users (people who hadn’t used the forecast in the past month or had no access) and “In Practice” users (people who had used the forecast at least once in the past month). There were 88 of 111 (79 %) survey responders that had access to the seizure risk forecast feature at the time of survey completion. Of these, 53 of 88 responders (60 %) viewed the forecast feature at least once within the prior month (“In Practice” users, n = 53). Responders who had never viewed the forecast (despite having access) and responders who did not have access were categorised as “In Theory” users (n = 58).

##### 2.2.2.1. User experiences and perspectives of seizure risk forecasting.

A series of questions targeting forecast usability was developed for user experience research of the mobile epilepsy management app. The questions were forced-choice format with the option to add a free text response. In Practice users were asked if they found the forecast accurate enough to be useful, what they had used it for and how they felt during high and low risk. In Theory users were asked how they would use a forecast and how they would expect to feel during high and low risk, if they had access to a forecast.

##### 2.2.2.2. Mood and adjustment.

All responders were also asked to complete standardised measures of mood (brEASI and NDDI-E questionnaires) and adjustment to illness (SERDAS and PHE questionnaires). Scoring procedures for mood and adjustment questionnaires can be found in Supplementary Appendix B. All mood and adjustment scores were calculated such that higher scores always represented the less favourable outcome (i.e., higher likelihood of anxiety, higher likelihood of depression, less adjustment to epilepsy and higher seizure-related disability).

###### 2.2.2.2.1. Brief Epilepsy Anxiety Survey Instrument (brEASI).

The brEASI [27] is an eight-item screening questionnaire for assessing symptoms of anxiety disorders. Higher scores indicate greater sympatomology and scores of *>*7 have been shown to have 84 % specificity and 76 % sensitivity for a dignosis of a generalised anxiety disorder in people with epilepsy.

###### 2.2.2.2.2. Neurological Disorders Depression Inventory in Epilepsy (NDDI-E).

The NDDI-E is a six-item screening measure for assessing symptoms of depression that do not overlap with the cognitive comorbidites of epilepsy or side-effects of anti-seizure medications [28]. Higher scores indicate greater sympatomology and scores of *>*15 have been shown to have 90 % specificity and 81 % sensitivity for a diagnosis of major depression in people with epilepsy.

###### 2.2.2.2.3. Adapted Patient Health Engagement (PHE) Scale.

The PHE scale is a nine-item instrument for assessing patient engagement in the healthcare process [29]. Questions from the PHE scale were adapted to examine patient engagement with the forecast feature. In particular, we drew on two questions and expanded them into eight separate items on a 5-point Likert scale (from *Strongly Agree* to *Strongly Disagree*) (see Supplementary Table 2). These eight statements were: I am very discouraged due to my illness, I feel anxious when I try to manage my illness, I feel I adjusted to my illness, I am generally optimist about my future and my health condition, I feel totally oppressed by my illness, I am upset when a new symptom arises, I feel I have accepted my illness, and I can give sense to my life despite my illness condition.

###### 2.2.2.2.4. Seizure Related Disability Assessment Scale (SERDAS).

The SERDAS [30] is a six-item seizure-related disability measure used to assess time lost to seizures and anti-seizure medication side effects. Three questions from the SERDAS were used in the current survey because they pertained to the fear and unpredictability of seizures.

#### 2.2.3. Semi-structured interviews

There were 21 participants who appeared ambivalent towards the use of the seizure risk forecasting app (i.e., they ticked “neither Agree not Disagree” on a Likert scale when asked if the forecast feature was accurate enough to be useful). Nine of these participants completed semi-structured interviews in order to gain insights into this “neutral stance” and explore their needs with regards to epilepsy management. We chose to include only neutral forecast users in the semi-structured interviews to understand areas for improvement without influence from overly skewed negative or positive opinions.

Interviews were conducted by four different clinic staff via video call and were approximately 60 min in length. All four staff members who conducted the interviews had experience or received training on qualitative interviewing techniques. Open-ended questions were utilised to gather rich, in-depth data. Prompts were utilised, if needed, to ask participants to expand upon answers, with a particular focus on seizure management. The full interview schedule can be found in Supplementary Appendix F.

Throughout the interviews, we monitored for thematic saturation and observed no significant emergence of new themes in the latter interviews. All interviews were transcribed verbatim using transcription software and were reviewed and verified by interviewers. A thematic content analysis was then used to analyse the data [31]. Data were coded inductively and assessed across all interviewees until thematic saturation was reached, noting that all themes and sub-themes were common to at least 3 out of 9 interviewees. A preliminary codebook emerged during the initial coding rounds (conducted by two people independently). This codebook was then refined through discussions between both coders to ensure consistency in applying the codes across the interviews.

### 2.3. Statistical analysis

**Fig. 2.**
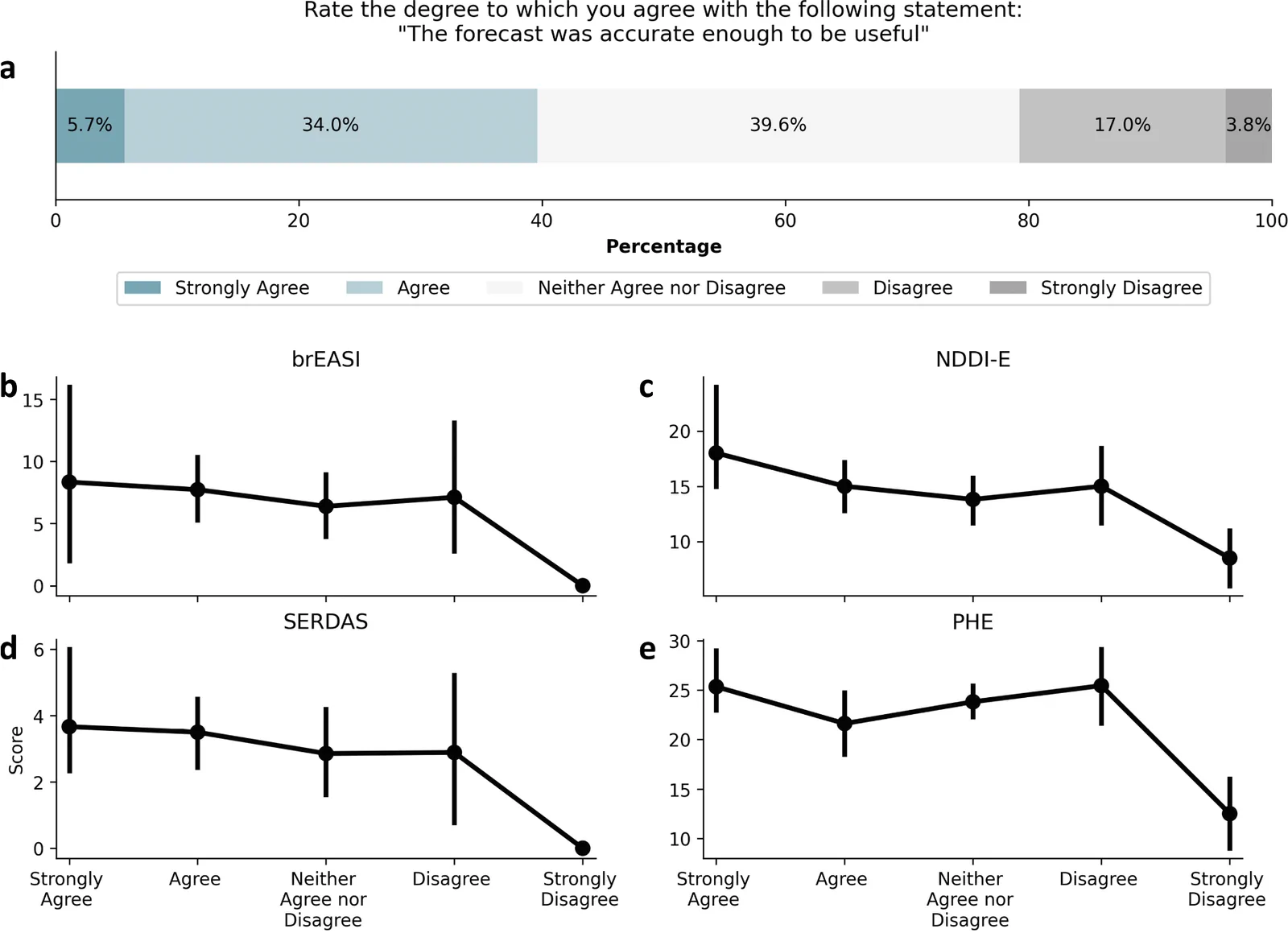
Perceived forecast accuracy and relationship to mood and adjustment scores reported by In Practice users. **a:** Percentage of responses to the statement, “The forecast was accurate enough to be useful.” **b–e:** Mood and association scores (mean ± standard deviation) of responders in comparison to their response to “The forecast was accurate enough to be useful.” Differences between mood/adjustment scores and responses were assessed using Mann-Whitney U tests with a Bonferroni correction for multiple comparisons.

Comparison of the basic demographic and clinical data between non-responder and responder (initial and follow-up) groups were assessed using Mann-Whitney U tests for continuous variables and χ^2^-tests for categorical variables (Table 1). Differences between mood/adjustment scores across responses to the perceived accuracy survey question (Fig. 2b–e) were assessed using Mann-Whitney U tests with a Bonferroni correction for multiple comparisons. To assess the correlations between forecast usage/risk responses and mood/adjustment scores, correlation tests (Phi correlation, Rank-biserial and Spearman correlation) were applied, depending on the continuous or categorical nature of the variables. Wilcoxon signed-rank tests were used to measure individual changes in mood scores between initial and follow-up timepoints. All statistical tests were non-parametric, as tested distributions were non-normal and/or on small sample sizes.

## 3. Results

### 3.1. Quantitative insights from survey

#### 3.1.1. Accuracy perspectives of the seizure risk forecast

In response to the question “Rate the degree to which you agree with the following statement: The forecast was accurate enough to be useful” (Fig. 2a), 39.7 % of In Practice users agreed (or strongly agreed) the feature was useful, 39.6 % were ambivalent (neither agreed nor disagreed) and 20.8 % disagreed (or strongly disagreed). While there was a trend for people with higher brEASI, NDDI-E and SERDAS scores to select a positive or neutral response to the usefulness question, these differences were not statistically significant (Fig. 2b–e).

Possible factors influencing perceived accuracy were investigated in Supplementary Appendix E. On a group level, perceived accuracy levels did not significantly change users’ usages or responses to low or high risk states. Like mood scores, there was a trend for seizure frequency to relate to perceived accuracy (higher seizure frequency in responders with lower perceived accuracy), although this was not significant.

#### 3.1.2. Behavioural response to the seizure risk forecast

Survey responders reported many uses (and expected uses) of the forecast feature, with “helping mental state” and “scheduling social and travel activities” being the most common uses identified (selected by 28 % and 17 %, respectively). Some responders (19 % of In Practice users) used the free text option to report using the forecast for “Other” reasons, such as “I feel more comfortable doing stressful things when I have less risk.” On the other hand, responders who chose not to use the forecast occasionally reported why they had made this choice, for instance “For the benefit of my mental state, I choose not to let it rule my day or what activities I’m planning to do, as I won’t let epilepsy rule my life nor hold me back.” Interestingly, there were no significant correlations between behavioural responses and perceived accuracy of the forecast (Supplementary Fig. 12).

The most common expected uses selected by the In Theory group were “helping mental state” (57 %) and “scheduling social and travel activities” (59 %). Almost half (~47 %) of In Theory responders noted a specific and personalised use case in free text, of which 74 % (35 % of all In Theory responders) mentioned interest in controlling treatment or medication in response to seizure risk forecasting.

**Fig. 3.**
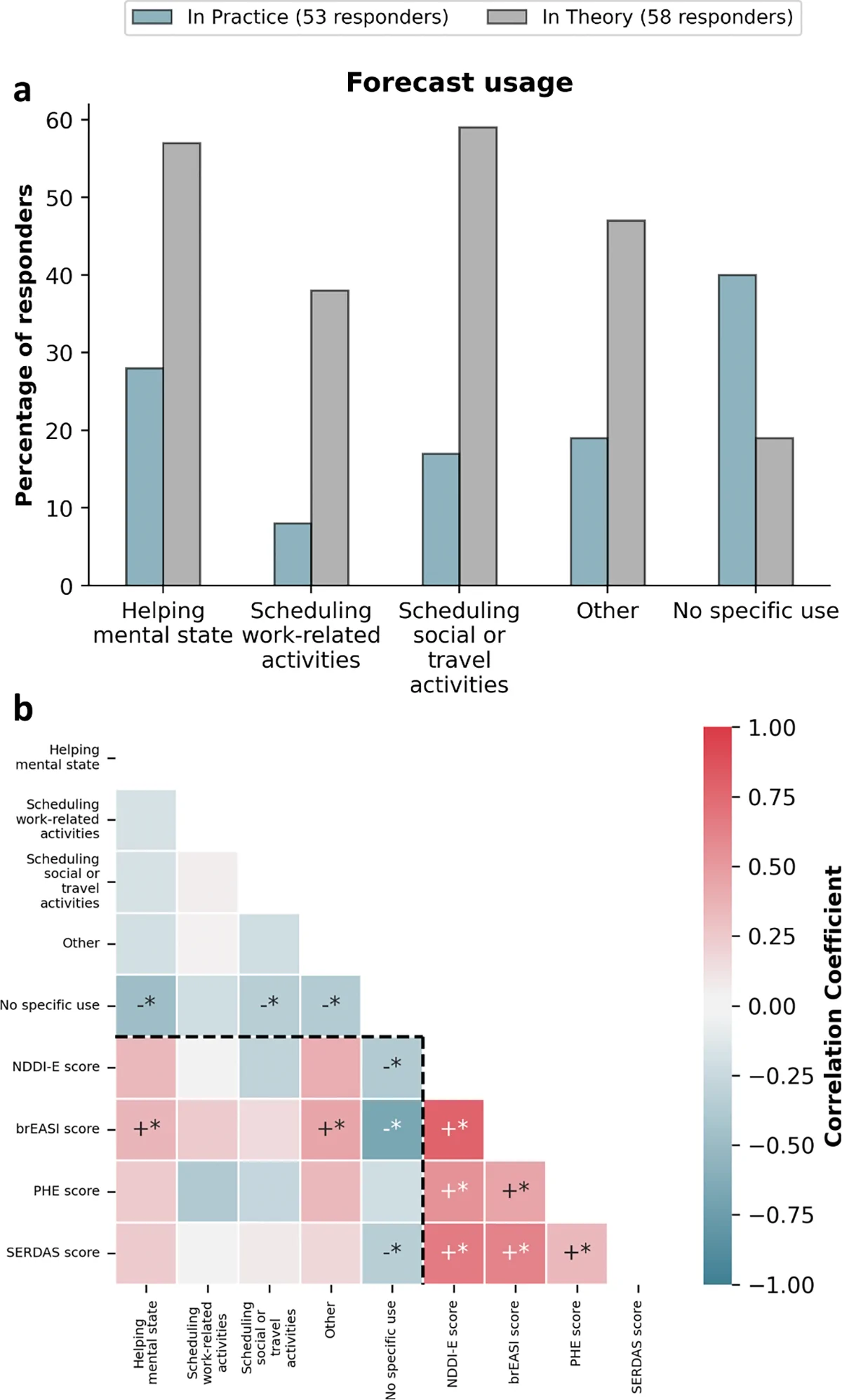
Forecast usage and relationship to mood and adjustment scores. **a:** Percentage of In Practice (blue) and In Theory (grey) users who responded to each tick box in response to a question about what the forecast was used for (or would be used for, in the case of In Theory users). In Practice users were asked “Have you ever changed your behaviour or activities because of the risk forecast and how (tick all that apply)?” and In Theory users were asked “In theory, if you had access to a seizure risk forecast, what would you use it for (tick all that apply)?” **b:** Correlation matrix to assess the relationships between answers to the forecasting usage question and mood and adjustment survey scores in the In Practice group. *Indicates significant correlation (p *<* 0.05), and the symbol (+ or -) next to the asterisk represents the direction of the correlation (positive or negative, respectively). Note that responses where users selected “No specific use” alongside other usage options (e.g., “Helping mental state”) were removed from the analysis. (For interpretation of the references to colour in this figure legend, the reader is referred to the web version of this article.)

Correlations between In Practice forecast feature usage and mood and adjustment scores were assessed in Fig. 3b. Significant positive correlations were detected between using the forecast to help mental state and “Other” uses and higher brEASI anxiety scores, suggesting that responders with more anxiety symptoms were more likely to use the forecast to manage their mental well-being. On the other hand, responders who did not identify a use had significantly lower (i.e. more favourable) brEASI, NDDI-E and SERDAS scores, indicating that individuals with lower mood scores and less disability were less likely to use the forecast to adapt their behaviour.

#### 3.1.3. Responses to forecasted risk condition

**Fig. 4.**
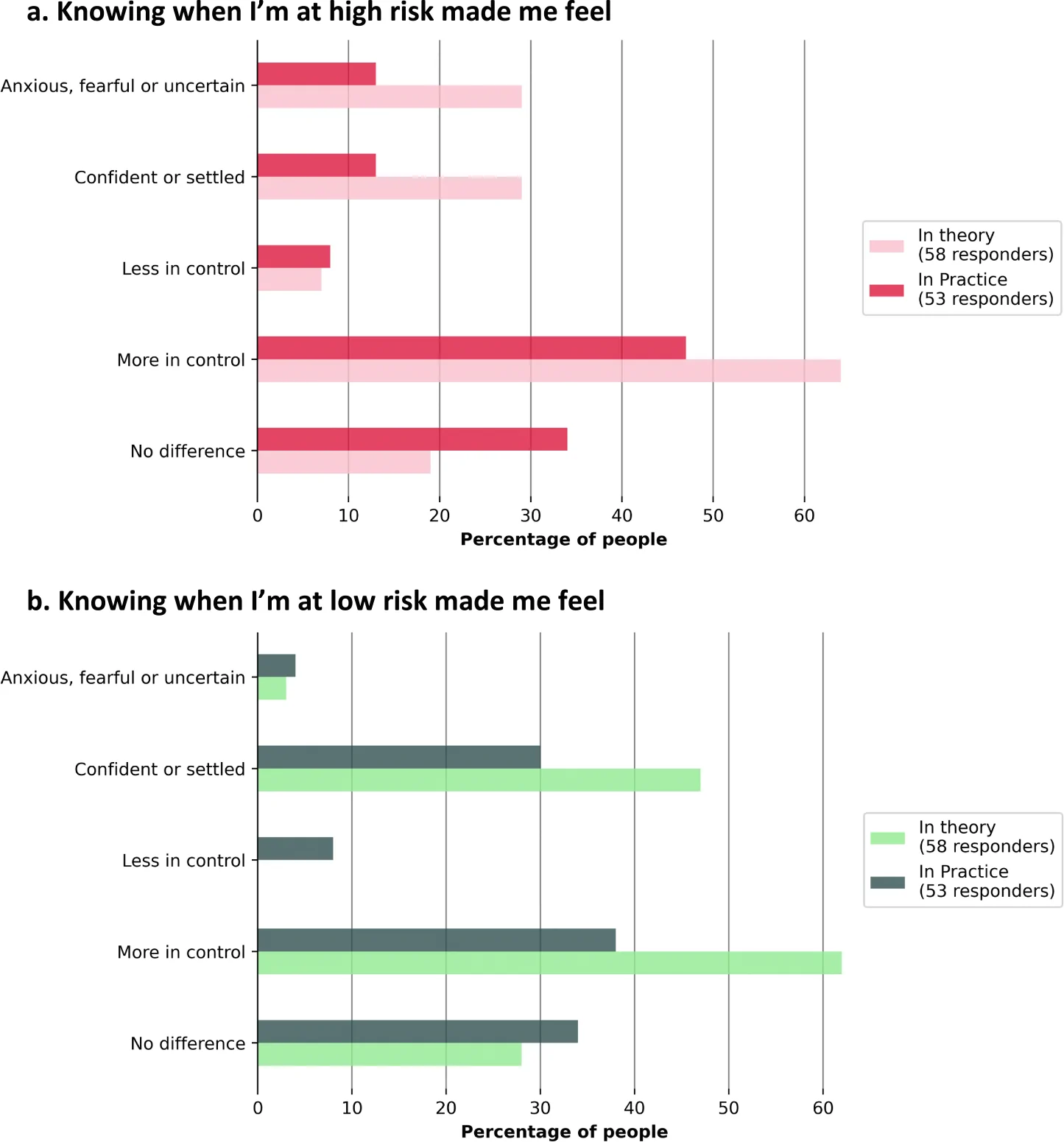
Impact of risk condition on mental wellbeing. In Practice and In Theory reported impact of **a:** high and **b:** low risk states. Note that users were able to select potentially contradicting responses (“More in control” and “Less in control”), as these may reflect genuine user experiences with complex emotions.

We also asked about the impact of risk condition (high risk vs. low risk) on mental state (Fig. 4). For both In Theory and In Practice groups, the most common responses (47–64 %) to high risk times were feelings of greater control. Similarly, the most common responses to low risk times were feeling more settled, and a greater sense of control and confidence.

Interestingly, 30 % of In Theory responders predicted feelings of anxiety, fear or uncertainty in response to high risk times in comparison to only 12 % of In Practice responders. Feeling less in control also occurred more commonly in the In Practice group compared to what was anticipated in the In Theory group (In Practice vs. In Theory: 8 % vs. 7 % during high risk and 8 % vs. 0 % during low risk, Fig. 4).

#### 3.1.4. Survey response changes over time

There were small changes in forecast accuracy perspectives between initial and follow-up timepoints (Supplementary Figs. 2 and 3), although the total number of surveyed users who agreed that the forecast was accurate enough to be useful (i.e., selecting “Agree” or “Strongly Agree”) remained constant between time points. As surveyed users gained experience with the forecast, views on forecast accuracy and utility appeared to become more polarised, with views often switching from a neutral stance to strongly agreeing or disagreeing that the forecast was useful. Small changes in forecast behavioural response and responses to risk condition were also observed between initial and follow-up timepoints (Supplementary Figs. 4 and 5).

Further analyses using mood scores (brEASI, NDDI-E) of responders can be found in Supplementary Appendix D, with no significant changes in mood scores in In Practice and In Theory groups observed between initial and follow-up timepoints.

### 3.2. Qualitative insights from semi-structured interviews

N = 9 survey responders who were ambivalent about the utility of the forecast were interviewed about their experiences with epilepsy management. Five broad themes emerged: (1) living with seizures: uncertainty and loss of independence, (2) feeling stuck, (3) desire to control and understand one’s epilepsy, (4) usefulness of technology in improving self-management, and (5) hope for the future (Table 2).

#### 3.2.1. Living with seizures: uncertainty and loss of independence

Most (7 of 9) interviewees shared their experiences of living with epilepsy, highlighting the pervasive uncertainty associated with both the condition and the occurrence of seizures. The apprehension of experiencing a seizure in potentially unsafe situations, such as while swimming, riding a horse, or climbing a ladder, was frequently mentioned. This concern extended to situations where the individual could potentially endanger others, such as holding a baby.

The fear of seizures interfering with daily activities often coincided with a sense of loss of independence, with many expressing the frustration of not being able to engage in activities they once enjoyed. Notably, 6 of 9 interviewees mentioned that being unable to drive impacted their sense of autonomy.

#### 3.2.2. Feeling stuck

Feeling stuck with the present situation was a theme expressed by all interviewees, even though most had been diagnosed with epilepsy more than 5 years prior. These frustrations particularly stemmed from a lack of definitive answers (usually regarding their treatment or diagnosis) and a desire for alternative methods to manage their seizures. Many inteviewees conveyed their ongoing efforts to explore different avenues for seizure management, often leading them to the risk forecasting app.

Frustration with anti-seizure medications was mentioned by 8 of 9 interviewees, often in regard to trialling numerous unsuccessful medications or experiencing undesirable side effects. The frustration was usually connected to their own experiences with the healthcare system’s approach to epilepsy treatment and/or a perceived lack of transparency from experts.

#### 3.2.3. Desire to control and understand one’s epilepsy

Most (8 of 9) interviewees emphasised the importance of gaining control and understanding their epilepsy. Specifically, 7 of 9 interviewees made statements consistent with the concept of ‘internal locus of control’, i.e. the belief in one’s ability to influence events in their life [32], which included sharing observations about patterns or triggers related to their seizures and how making changes based on these insights had proven helpful.

Additionally, 8 of 9 interviewees expressed the benefits of, or a desire for, increased self-knowledge and data insights into their condition. In particular, using technology (e.g., an app or device) that provided insights into their epilepsy appeared to improve their internal locus of control.

#### 3.2.4. Usefulness of technology in improving self-management

The usefulness of technology in improving self-management of epilepsy and seizures was mentioned by 8 of 9 interviewees, both in regard to seizure forecasting and other technologies. An app to log epilepsy-related information, such as seizure times and details, medications, and changes in feelings or symptoms, was often mentioned as an important component of epilepsy management, for the patient and for their neurologist.

Additionally, 8 of 9 interviewees expressed opinions on the current seizure forecasting app, recognising its usefulness or anticipated usefulness with future iterations. Some participants had already modified their activities, such as alcohol consumption, based on the app’s insights, while others, though sceptical about its accuracy, remained hopeful for future improvements that could potentially allow them to adapt their behaviour.

#### 3.2.5. Hope for the future

A sense of hope for improved epilepsy management in the future was expressed by 5 of 9 interviewees, marked by optimism regarding ongoing research and upcoming advancements in seizure management. These positive sentiments revolved around the contributions of clinicians and researchers, as well as the current and anticipated developments in seizure management technology.

## 4. Discussion

In recent years, seizure risk forecasting has emerged as a clinical tool to address seizure unpredictability and improve quality of life for people living with epilepsy [25,33]. While remarkable strides have been made in the development of seizure forecasting models [4–7,9,10,12,14,34,35], very little is understood about the real-world impact of such a technology, particularly on patient behaviour, quality of life and effectiveness in reducing seizure unpredictability. This mixed methods study addresses this gap in understanding, by examining user behaviour changes and perspectives of a seizure risk forecast tool, as well as links between user experience/perspectives and mood and adjustment to epilepsy.

### 4.1. User experiences and perspectives of the seizure risk forecast

We found in our cohort that the seizure risk forecast feature of the mobile epilepsy management app was adopted by most (~60 %) surveyed users and perceived as accurate enough to be useful to almost half of surveyed users. This is on par with one previous community survey study, which found that 76 % of people would use a seizure forecasting device but only one-third believed a device could predict their seizures [17]. While the novelty of this study limited a direct benchmark comparison, our findings corroborate previous findings that seizure forecasting is important to the epilepsy community and that most people are motivated to use a device that predicts their seizure risk [17,25].

We found that more than half of surveyed users reported at least one behavioural change in response to the seizure risk forecast, such as helping mental state and scheduling social or travel activities. Behavioural changes were significantly less likely amongst responders with lower mood and adjustment scores but significantly more likely for certain behaviours among people with higher anxiety symptoms, collectively suggesting that seizure forecasts are more useful to people with greater mood disorder symptoms. While there was a trend for people with lower mood and adjustment scores to disagree with the forecast’s accuracy and usefulness, this seemed to be driven by a small number of users, and thus needs to be established in a larger cohort before any conclusions can be drawn. Understanding the needs of individuals with fewer mood symptoms that are more well-adjusted to their condition may also require a more nuanced approach (e.g., through targeted interviews). While we did not observe any changes in mood/ adjustment scores within the study period (i.e., at baseline compared to follow-up) in this work, it is possible that future studies may see improvements in mood/adjustment scores with prolonged forecast use in controlled environments. Such studies should pay particular attention to anxiety disorder symptom changes, since we observed that people with higher anxiety scores were significantly more likely to use the forecast for helping mental state.

The impact of risk condition (high vs. low risk) on mental state was also examined. Feelings of greater control and increased confidence were the most common responses to both high and low risk states. These positive experiences were reported by surveyed users who both agreed and were ambivalent in response to the perceived accuracy question, indicating that it is possible to experience positive feelings from viewing a seizure forecast even without confidence in its accuracy. Nonetheless, a common concern amongst epilepsy researchers and clinicians is that seizure forecasting will cause feelings of anxiety, particularly during higher risk periods. This was observed within the cohort, albeit to a lesser extent than expected (12 % In Practice vs. 30 % In Theory). While we did not identify any significant associations between mood/adjustment scores and responders who felt anxious, predicting potential anxious responses may be important in future to minimise possible negative side effects of seizure forecasts.

Furthermore, our findings demonstrate a clear ‘intention-behaviour gap’ [21] between stated willingness and actual behaviour change. While both In Practice and In Theory groups expressed similar trends in desired behaviour, discrepancies emerged in the proportion of responders who translated those perceived benefits into action. This gap can be largely attributed to user confidence in the forecast’s accuracy; i.e., those with lower confidence were less likely to adjust their behaviour and often reported feeling “no difference” during high and low-risk states. These discrepancies underscore the necessity of incorporating real-world user perspectives into seizure forecast development to ensure systems are tailored to address actual user needs.

### 4.2. Addressing uncertainty

Uncertainty is often ranked as the most difficult part of living with epilepsy [2,3,19]. In line with previous work, living with uncertainty as part of having a diagnosis of epilepsy was a theme expressed by 7 of 9 of the interviewees. For these people, not only did the fear of having a seizure interrupt normal life, but it was associated with psychosocial restrictions and affected their sense of independence.

Most interviewees (8 of 9) therefore emphasised the importance of gaining control and understanding their epilepsy, likely to help combat feelings of uncertainty. The interviews gave us insight into the idea of gaining control, suggesting that this could be achieved, to a degree, through observing patterns and triggers in one’s seizures, and explains a shared desire for more self-knowledge and data insights into their condition. These discoveries suggest that feelings of uncertainty experienced by people with epilepsy are not only associated with the unpredictability of seizure occurrence, but also with the extent to which they believe they have control over and can influence events in their life (i.e., internal locus of control). Higher internal locus of control has previously been linked to perceived control of seizure occurrence [36], knowledge and understanding of the condition [37] and improved social support networks [38] in people with epilepsy.

Amongst interviewees, the use of technology (e.g., an app or device) that provided insights into their condition appeared to improve sense of control, and this was often discussed with reference to the seizure forecasting app, despite the interviewees’ reservations about the accuracy of the forecast. This observation is in line with our survey responses, which highlighted that feelings of greater control and confidence were the most common reactions to forecasted risk states. These findings, also revealed in a previous study [24], indicate that simply providing a tool that facilitates a greater understanding of one’s epilepsy and seizure patterns could be helpful to reduce uncertainty and improve internal locus of control.

### 4.3. Limitations and future work

This study had limitations. First, the relatively low survey response rate (9.5 %), although consistent with previous response rates [18,26], meant that many of the relationships investigated in this work were assessed on small cohort sizes (e.g., under 50 people), which may lead to difficulty in detecting significant relationships between variables, particularly factors related to perceived accuracy (e.g., seizure frequency and mood scores). To mitigate this, all significance tests used in this work were non-parametric. Furthermore, as with any convenience sampling study, survey responders may exhibit bias, such as selection or response bias. Response bias, where responders answer questions inaccurately or incompletely due to factors such as memory recall or comprehension, may be a confounding variable in this work; however, controlled, prospective clinical trials are necessary to address many of these limitations.

This study has two potential limitations related to selection bias. Survey responders, recruited through a convenience sampling method (email survey), may not represent the entire app user base. Engagement, technical expertise, app usage patterns, disease severity and prior forecasting experience are just some factors that could influence survey participation. While our analysis of clinical and demographic data suggests responders were within the target population (people with epilepsy) and does not indicate differences in disease severity (number of anti-seizure medications and disability) between non-responder and responder groups, future studies should collect data on engagement and app usage for a more robust assessment of selection bias. However, it is important to note that our analysis of forecast performance between responders and non-responders did not reveal significant differences, suggesting some generalisability of responders’ experiences. Additionally, the semi-structured interviews only included participants with neutral feedback on the seizure forecast. This approach allowed us to target the largest responder sub-group to better understand the most needed improvements that could be made to seizure forecasts without the influence of potential biases or overly skewed positive and negative opinions. However, to understand the full spectrum of user experiences, particularly regarding significant concerns and the role of factors like mood and seizure disability in perceived forecast accuracy and usability, future studies should look to interview participants with all forecast perspectives.

Second, many of the measurements and questions around forecast accuracy were subjective; i.e., we did not compare perceived accuracy to true forecast accuracy. This aligns with the understanding that defining outcomes relevant to patients is crucial for developing effective patient support tools [15,39] and that patients are usually willing to accept devices with less than perfect accuracy [15,17,19]. Moreover, the forecaster model used in the current work [10] was previously shown to perform akin to state-of-the-art seizure forecasting models [4,6,9,14,40]. Nonetheless, true forecast accuracy compared to perceived accuracy should be investigated in future work, and ideally in a controlled environment, as access to a seizure forecast may change behaviour and/or seizure frequency (e.g., through measures taken by patients to reduce seizure likelihood during forecasted high risk periods) [33].

Third, since this study was designed to be brief and simple for forecast users to complete, we compromised on the length of the survey, meaning that there are still many unanswered questions when it comes to investigating whether seizure forecasting can mitigate feelings of anxiety and fear without encouraging risk-taking behaviours. Nonetheless, one important observation made in this work was that people seemed to be able to self-regulate their response to the seizure forecast system. For instance, around 40 % of the In Practice cohort simply chose not to use the forecast. Moreover, many people who were undecided on the usefulness (in terms of accuracy) of the forecast at baseline had made their mind up at the follow-up timepoint, suggesting that most surveyed users were confident in making their own decisions about the accuracy and utility of the forecast. Participants’ autonomy was also made clear in interviews and personalised responses to the survey, e.g., “I choose not to let it rule my day or what activities I’m planning to do”. These findings highlight an important potential benefit of non-invasive systems, whereby a forecast presented in a mobile application can potentially be ignored or uninstalled by a user, which differs from the only previous prospective study of this nature where the device was physically implanted in the patient [5], and resulted in more dramatic effects on users’ sense of control and sense of self [22]. Additionally, while the psychological and emotional impact of implanted medical devices compared to non-invasive systems is yet to be directly compared, non-invasive systems offer several advantages. They are associated with reduced risk of stigmatisation [16,23], particularly when using consumer-grade smartwatches, are often less expensive and less risky, and may reduce cognitive load [41] and the potential for self-estrangement compared to brain implantable devices [42]. Notably, for seizure forecasting applications, non-invasive systems are preferred by patients [16,19,20,23,24], although invasive systems are superior in terms of seizure identification [5] and, consequently, forecasting performance [4].

Future clinical trials are required to address the limitations in this study, as well as further questions in regard to patient response to seizure risk forecasting [33]. These trials should look to observe potential changes in patient behaviour as well as quality of life, seizure rates, mood scores and stress levels, across a variety of different forecast designs and horizons and compared to controlled cohorts (e.g. by using covert or random forecasts). This study also revealed the importance of quantifying changes in internal locus of control as a metric of forecast success, as this trait was not only important to people with epilepsy but also seemed to be positively impacted with access to a seizure forecaster.

## 5. Conclusions

This is the first study to investigate user experiences and perspectives of a non-invasive seizure risk forecast mobile app for people with epilepsy. Our preliminary findings indicate that most people living with epilepsy express interest in monitoring their seizure risk forecast and occasionally utilising it to modify their behaviours, particularly people with greater mood disorder symptoms. Findings also point to the potential for improved self-knowledge and a greater sense of control that could be fostered through the use of seizure forecaster apps. This may have a positive impact on the quality of life of people living with epilepsy and warrants further exploration.

## Supplementary Material

Supplement

## Figures and Tables

**Table 1:** Clinical and demographic data in survey responder and non-responder groups.

	Responders (mean ± standard deviation)	Non-responders (mean ± standard deviation)	p-value

Seizure frequency (per day)	Initial (n = 51): 0.3693 ± 1.217 Follow-up (n = 19): 0.7212 ± 1.925	0.2854 ± 0.5604 (n = 611)	Initial: p = 0.2378 Follow-up: p = 0.3269
Age (years)	Initial (n = 71): 41.13 ± 18.00 Follow-up (n = 24): 43.22 ± 21.31	32.77 ± 18.33 (n = 699)	Initial: 0.0002* Follow-up: 0.0230*
Gender (male: female)	Initial: 43:31 Follow-up: 15:11	486:219	Initial: p = 0.1257 Follow-up: p = 0.0149*
Epilepsy diagnosis (yes:unknown)	Initial: 65:7 Follow-up: 24:1	510:187	Initial: p = 0.0024* Follow-up: p = 0.0201*
Epilepsy syndrome (focal: generalized)	Initial: 45:14 Follow-up: 16:5	305:166	Initial: p = 0.1063 Follow-up: p = 0.3995
Number of ASMs	Initial (n = 42): 1.93 ± 1.24 Follow-up (n = 15): 2.07 ± 1.33	1.75 ± 1.41 (n = 424)	Initial: p = 0.8415 Follow-up: p = 0.9840
Carer (yes:no)	Initial: 60:9 Follow-up: 21:3	413:248	Initial: p < 0.0001* Follow-up: p = 0.0125*
Disability (yes:no)	Initial: 12:57 Follow-up: 6:18	152:493	Initial: p = 0.2465 Follow-up: p = 0.8710

Differences in seizure frequency were investigated between responders and non-responders with seizure diaries of at least 30 days.

Note that age, gender, epilepsy diagnosis, epilepsy syndrome, number of anti-seizure medications (ASMs), carer and disability data is only available for patients with a linked EEG study and completed referral form section relevant to the clinical data.

Statistical tests are described in Section 2.3.

ASMs = Anti-seizure medications.

* p < 0.05.

**Table 2:** Commonly reported themes when discussing managing epilepsy.

Theme	N (%)	Subtheme	Examples

Living with seizures: uncertainty and loss of independence	7 (78 %)	Fear of having a seizure	**S1:** “I better not, I better not get up on the ladder. I better not walk out the door. You know, my wife and I go to the grocery store. I’m afraid I may have a seizure.” **S9:** “And so I get worried I guess when we go out and I don’t know, I don’t really know when they’re going to happen”
	7 (78 %)	Loss of independence	**S8:** “It’s so unpredictable. I’m not supposed to drive, that’s hard.” **S6:** “Now my whole persona has changed, my identity has changed, my independence has changed.”
Feeling stuck	6 (67 %)	Wanting another way to manage seizures	**S6:** “I’m not anti-medication, but it always seems that’s the answer to everything. Medication, medication, medication. There’s got to be other ways of managing seizures”
	9 (100 %)	Frustrations with lack of direction	**S1:** “Seems like the research part of things is a lot slower than the actual doing of things. It’s frustrating for me.” **S9:** “And I think that’s the way my neurologist sees it is that if they’re not life-threatening then we just make do with what we’re doing.” **S2:** “I mean, I’ve had so many EEGs and none of them have come up with anything, you know, they just can’t come up with any, with any answers, you know, and it’s frustrating.”
Desire to control and understand one’s epilepsy	7 (78 %)	Internal locus of control	**S5:** “[Re the after-effects of a seizure] I don’t want to be a victim. I don’t want to sit around and do nothing. I want to kind of keep going and it makes you have more control over it.” **S3:** “Because I like to manage my own. Myself. I, I like to know what’s going on with my body.”
	8 (89 %)	Benefits of increased self-knowledge and data insights	**S5:** “And I guess that’s why I’m on there because it gives me a little bit of insight that I don’t have from anywhere else.” **S4:** “I hate being left in the dark on this sort of stuff.” **S6:** “You know, I can’t say this enough. Knowledge is power. If you don’t have the knowledge, knowledge creates fear. Fear creates doubts. Doubts creates chaos.”
Usefulness of technology in improving self-management	7 (78 %)	Benefits of current technology for seizure management	**S5:** “I think tech is really, really important when it comes to managing healthcare and managing epilepsy.” **S8:** “I tried to write things down on occasions, but then I got too lazy, I suppose. I find this really easy because you’ve always got your phone with you. It’s so easy.”
	8 (89 %)	Current and anticipated usefulness of forecasting	**S2:** “But I think it is good. I think it’s useful. It also enables me to make plans, if you like, and to make sure that wherever I am, that I make sure that I’m safe.” **S5:** “It’s not always correct but it just gives you good indication of, okay, maybe I’ll stay home tonight or maybe I’ll take somebody with me that knows what to do. So Yeah. Or not going to have 10 drinks tonight, I’ll have two glasses of wine instead.” **S7:** “The, the nice thing is, if, if you know what your cycles are, then you can kind of help plan when they might happen.”
Hope for the future	3 (33 %)	Optimistic about ongoing research	**S5:** “[Re the forecasting app] It just gives you that little bit of hope around people investing in something that impacts you”
	3 (33 %)	Hoping for better seizure management	**S3:** “[Re increase in medication dosage] So I’m hoping that might bring some of the seizures down.” **S8:** “[Re the forecasting app] I was just hopeful that it would work... So you’ll be able to predict and manage around it. Yeah. And help people worse off than what I am more so. Yeah. Will be wonderful.”

## Data Availability

Deidentified data used in this work will be made available upon reasonable request. Requests for access to these data should be directed to Rachel E. Stirling (rachelstirling1@gmail.com).

## Appendix A. Supplementary material

Supplementary data to this article can be found online at https://doi.org/10.1016/j.yebeh.2024.109876.

## References

[1] Kwan P, Brodie MJ. Early identification of refractory epilepsy. N Engl J Med 2000; 342:314–9. 10660394 10.1056/NEJM200002033420503

[2] Arthurs S, Zaveri HP, Frei MG, Osorio I. Patient and caregiver perspectives on seizure prediction. Epilepsy Behav 2010;19:474–7. 20851054 10.1016/j.yebeh.2010.08.010

[3] Foundation Epilepsy. Ei2 community survey. Landover, MD: Epilepsy Foundation; 2016.

[4] Proix T, Truccolo W, Leguia MG, Tcheng TK, King-Stephens D, Rao VR, Forecasting seizure risk in adults with focal epilepsy: a development and validation study. Lancet Neurol 2021;20:127–35. 10.1016/S1474-4422(20)30396-3. 33341149 PMC7968722

[5] Cook MJ, O’Brien TJ, Berkovic SF, Murphy M, Morokoff A, Fabinyi G, Prediction of seizure likelihood with a long-term, implanted seizure advisory system in patients with drug-resistant epilepsy: a first-in-man study. Lancet Neurol 2013;12:563–71. 10.1016/S1474-4422(13)70075-9. 23642342

[6] Maturana MI, Meisel C, Dell K, Karoly PJ, D’Souza W, Grayden DB, Critical slowing down as a biomarker for seizure susceptibility. Nat Commun 2020;11: 2172. 10.1038/s41467-020-15908-3. 32358560 PMC7195436

[7] Karoly PJ, Ung H, Grayden DB, Kuhlmann L, Leyde K, Cook MJ, The circadian profile of epilepsy improves seizure forecasting. Brain 2017;140:2169–82. 10.1093/brain/awx173. 28899023

[8] Chen Z, Grayden DB, Burkitt AN, Seneviratne U, D’Souza WJ, French C, Spatiotemporal patterns of high-frequency activity (80–170 Hz) in long-term intracranial EEG. Neurology 2020. 10.1212/WNL.0000000000011408 33361261

[9] Stirling RE, Grayden DB, D’Souza W, Cook MJ, Nurse E, Freestone DR, Forecasting seizure likelihood with wearable technology. Front Neurol 2021;12: 704060. 34335457 10.3389/fneur.2021.704060 PMC8320020

[10] Xiong W, Stirling RE, Payne DE, Nurse ES, Kameneva T, Cook MJ, Forecasting seizure likelihood from cycles of self-reported events and heart rate: a prospective pilot study. EBioMedicine 2023;93. 10.1016/j.ebiom.2023.104656 PMC10300292 37331164

[11] Duun-Henriksen J, Baud M, Richardson MP, Cook M, Kouvas G, Heasman JM, A new era in electroencephalographic monitoring? Subscalp devices for ultra–long-term recordings. Epilepsia 2020;61:1805–17. 32852091 10.1111/epi.16630

[12] Stirling RE, Maturana MI, Karoly PJ, Nurse ES, McCutcheon K, Grayden DB, Seizure forecasting using a novel sub-scalp ultra-long term EEG monitoring system. Front Neurol 2021;12:713794. 34497578 10.3389/fneur.2021.713794 PMC8419461

[13] Stacey WC. Seizure prediction is possible-now let’s make it practical. EBioMedicine 2018;27:3–4. 10.1016/j.ebiom.2018.01.006. 29321121 PMC5828555

[14] Nasseri M, Pal Attia T, Joseph B, Gregg NM, Nurse ES, Viana PF, Ambulatory seizure forecasting with a wrist-worn device using long-short term memory deep learning. Sci Rep 2021;11:21935. 34754043 10.1038/s41598-021-01449-2 PMC8578354

[15] Janse SA, Dumanis SB, Huwig T, Hyman S, Fureman BE, Bridges JFP. Patient and caregiver preferences for the potential benefits and risks of a seizure forecasting device: a best–worst scaling. Epilepsy Behav 2019;96:183–91. 10.1016/j.yebeh.2019.04.018. 31150998

[16] Bruno E, Simblett S, Lang A, Biondi A, Odoi C, Schulze-Bonhage A, Wearable technology in epilepsy: the views of patients, caregivers, and healthcare professionals. Epilepsy Behav 2018;85:141–9. 29940377 10.1016/j.yebeh.2018.05.044

[17] Grzeskowiak CL, Dumanis SB. Seizure forecasting: patient and caregiver perspectives. Front Neurol 2021;12:717428. 34616352 10.3389/fneur.2021.717428 PMC8488220

[18] Chiang S, Moss R, Black AP, Jackson M, Moss C, Bidwell J, Evaluation and recommendations for effective data visualization for seizure forecasting algorithms. JAMIA Open 2021;4:ooab009. 33709064 10.1093/jamiaopen/ooab009 PMC7935496

[19] Schulze-Bonhage A, Sales F, Wagner K, Teotonio R, Carius A, Schelle A, Views of patients with epilepsy on seizure prediction devices. Epilepsy Behav 2010;18: 388–96. 20624689 10.1016/j.yebeh.2010.05.008

[20] Hoppe C, Feldmann M, Blachut B, Surges R, Elger CE, Helmstaedter C. Novel techniques for automated seizure registration: patients’ wants and needs. Epilepsy Behav 2015;52:1–7. 26366675 10.1016/j.yebeh.2015.08.006

[21] Sheeran P Intention—behavior relations: a conceptual and empirical review. Eur Rev Soc Psychol 2002;12:1–36.

[22] Gilbert F, Cook M, O’Brien T, Illes J. Embodiment and estrangement: results from a first-in-human “Intelligent BCI” trial. Sci Eng Ethics 2017:1–14. 10.1007/s11948-017-0001-5. PMC6418065 29129011

[23] Nasseri M, Nurse E, Glasstetter M, Bottcher S, Gregg NM, Laks Nandakumar A, Signal quality and patient experience with wearable devices for epilepsy management. Epilepsia 2020;61:S25–35. 32497269 10.1111/epi.16527

[24] Biondi A, Simblett SK, Viana PF, Laiou P, Fiori AM, Nurse E, Feasibility and acceptability of an ultra-long-term at-home EEG monitoring system (EEG@ HOME) for people with epilepsy. Epilepsy Behav 2024;151:109609. 38160578 10.1016/j.yebeh.2023.109609

[25] Dumanis SB, French JA, Bernard C, Worrell GA, Fureman BE. Seizure forecasting from idea to reality. outcomes of the my seizure gauge epilepsy innovation institute workshop. ENEURO.0349–17.2017 eNeuro 2017;4. 10.1523/ENEURO.0349-17.2017. PMC5744646 29291239

[26] Chiang S, Moss R, Patel AD, Rao VR. Seizure detection devices and health-related quality of life: a patient-and caregiver-centered evaluation. Epilepsy Behav 2020; 105:106963. 32092459 10.1016/j.yebeh.2020.106963

[27] Scott AJ, Sharpe L, Thayer Z, Miller LA, Hunt C, MacCann C, Design and validation of two measures to detect anxiety disorders in epilepsy: the Epilepsy Anxiety Survey Instrument and its brief counterpart. Epilepsia 2019;60:2068–77. 31560136 10.1111/epi.16348

[28] Friedman DE, Kung DH, Laowattana S, Kass JS, Hrachovy RA, Levin HS. Identifying depression in epilepsy in a busy clinical setting is enhanced with systematic screening. Seizure 2009;18:429–33. 19409813 10.1016/j.seizure.2009.03.001

[29] Graffigna G, Barello S, Bonanomi A, Lozza E. Measuring patient engagement: development and psychometric properties of the Patient Health Engagement (PHE) Scale. Front Psychol 2015;6:274. 25870566 10.3389/fpsyg.2015.00274 PMC4376060

[30] French JA, Porter R, Varner J, Schulz A-L, Zhang Y, Martin M. Baseline Seizure related disability assessment scale (SERDAS) scores in an observational study of brivaracetam (378). AAN Enterprises 2020.

[31] Coleman H, McIntosh A, Wilson SJ. Identifying the trajectory of social milestones 15–20 years after epilepsy surgery: realistic timelines for postsurgical expectations. Epilepsia Open 2019;4:369–81. 31440719 10.1002/epi4.12341 PMC6698676

[32] Boddu VK, Rebello A, Chandrasekharan SV, Rudrabhatla PK, Chandran A, Ravi S, How does “locus of control” affect persons with epilepsy? Epilepsy Behav 2021;123:108257. 34425327 10.1016/j.yebeh.2021.108257

[33] Baud MO, Proix T, Gregg NM, Brinkmann BH, Nurse ES, Cook MJ, Seizure forecasting: bifurcations in the long and winding road. Epilepsia 2022. 10.1111/epi.17311 PMC9681938 35604546

[34] Viana PF, Pal Attia T, Nasseri M, Duun-Henriksen J, Biondi A, Winston JS, Seizure forecasting using minimally invasive, ultra-long-term subcutaneous electroencephalography: individualized intrapatient models. Epilepsia 2022. 10.1111/epi.17252 PMC9547037 35395101

[35] Brinkmann BH, Wagenaar J, Abbot D, Adkins P, Bosshard SC, Chen M, Crowdsourcing reproducible seizure forecasting in human and canine epilepsy. Brain 2016;139:1713–22. 10.1093/brain/aww045. 27034258 PMC5022671

[36] Wolf P, Lin K, Mameniškiené R, Walz R. Does epilepsy have an impact on locus of control? Front Psychol 2020;11:2251. 33013587 10.3389/fpsyg.2020.02251 PMC7509065

[37] Tieffenberg JA, Wood EI, Alonso A, Tossutti MS, Vicente MF. A randomized field trial of ACINDES: a child-centered training model for children with chronic illnesses (asthma and epilepsy). J Urban Health 2000;77:280–97. 10856009 10.1007/BF02390539 PMC3456130

[38] Amir M, Roziner I, Knoll A, Neufeld MY. Self-efficacy and social support as mediators in the relation between disease severity and quality of life in patients with epilepsy. Epilepsia 1999;40:216–24. 9952270 10.1111/j.1528-1157.1999.tb02078.x

[39] Kuhlmann L, Lehnertz K, Richardson MP, Schelter B, Zaveri HP. Seizure prediction — ready for a new era. Nat Rev Neurol 2018;14:618–30. 10.1038/s41582-018-0055-2. 30131521

[40] Karoly PJ, Cook MJ, Maturana M, Nurse ES, Payne D, Brinkmann BH, Forecasting cycles of seizure likelihood. Epilepsia 2020;61:776–86. 32219856 10.1111/epi.16485

[41] Chee L, Valle G, Preatoni G, Basla C, Marazzi M, Raspopovic S. Cognitive benefits of using non-invasive compared to implantable neural feedback. Sci Rep 2022;12:16696. 36202893 10.1038/s41598-022-21057-y PMC9537330

[42] Postan E Narrative devices: Neurotechnologies, information, and self-constitution. Neuroethics 2021;14:231–51. 34721724 10.1007/s12152-020-09449-1 PMC8549978

