# Supplementary material for: User experience of a seizure risk forecasting app: A mixed methods investigation
Source: Epilepsy Behav. Author manuscript; Available in PMC 2026 Aug 31. (PMC13529357; doi:10.1016/j.yebeh.2024.109876)
Supplement: Supplementary file 1 [file NIHMS2202128-supplement-Supplement.docx]

# Supplement

## Appendix A: Seizure risk forecast algorithm design

The forecasting algorithm was based on previous research demonstrating the cyclical nature of seizure timing in most people with epilepsy (1–5) and the link between heart rate cycles and seizure timing (6–8). In brief, two algorithms (event-based and heart rate) were built to forecast seizure risk. For users without a smartwatch, only the event-based algorithm was deployed; for users with a linked smartwatch (Fitbit, Google), both algorithms were deployed and then combined into one forecast using a logistic regression model. Forecasts were retrained every 7 days or when a new event was reported.

In the event-based algorithm, seizure cycles were extracted and the two strongest plus a circadian cycle were used, provided they were above a phase locking value (PLV) of 0.3 (herein denoted as significant cycles). The PLV is a measure of the strength of the resultant vector and cycle, where 1 represents perfect alignment of seizure times to a specific phase and 0 represents perfectly uniform distribution around the cycle (i.e., seizures occurred at all phases of the cycle equally) (4). For each significant cycle, a Von Mises distribution of seizure risk based on historical phases of seizure occurrence was modelled. Past and future likelihoods for individual cycles were then generated by mapping the Von Mises distribution onto the cycle in the time domain. Individual cycle likelihoods (past and future) were combined using log-odds to produce a single likelihood value every hour. Daily likelihoods were calculated as the mean of future hourly likelihoods over a 24-hour period.

In the heart rate algorithm, raw heart rate (beats per minute, BMP) was down-sampled from 0.2 Hz to an average BPM value every 5 min (1/300 Hz) and missing data was interpolated with the global mean of the remaining timeseries. A Morlet wavelet transform was used to extrapolate periodic information in the signal. Significant peaks (above the time-averaged significance test threshold (9)) in the periodogram were considered as potential cycles. In line with previous work (6,7), the down sampled timeseries was filtered at these peaks (±33% of the peak period to account for phase shifts) using a Butterworth bandpass filter and phases were extracted using a Hilbert transform. Reported seizure times were mapped to heart rate cycle phases and the PLV was calculated for each cycle. Cycles with PLVs greater than 0.3 were considered significant and were projected into the future using a linear regression model of past phases and past timestamps. Future individual cycle likelihoods were derived from the model on an hourly timescale and combined using a log-odds model, similar to the event-based algorithm. Daily likelihoods were calculated as the mean of future hourly likelihoods over a 24-hour period.

In situations where both algorithms returned a forecast, event-based and heart rate algorithms were combined using a logistic regression model with coefficients trained on hourly past likelihoods from each algorithm (independent variables) and seizure hour times (dependent variable).

Hourly and daily likelihoods were converted to risk scores (1=low, 2=medium, 3=high) using low-medium and medium-high risk thresholds. These thresholds were computed using an optimisation algorithm described in previous work (7,8,10).

Notably, forecasts were only shown to users if at least one cycle (seizure cycle or heart rate cycle) had a PLV greater than the PLV threshold,

$\mathrm{PLV}_{\mathrm{threshold}} =0.3+0.02*(20-N) \mathrm{for} N<20 \mathrm{and} \mathrm{PLV}_{\mathrm{threshold}}=0.3 \mathrm{for} N\geq20$,	[1]

where $N$ is the number of reported seizures.

## To provide the most performant forecast for users, the algorithm was incrementally improved during the time in which this study was run; however, changes were minor and did not substantially affect the outcomes of this work.

## Appendix B: Survey design and scoring systems

**Supplementary Figure 1.** **Survey design, comprising two main sections: seizure risk forecasting perspectives, and mood and adjustment questionnaires.** Qu represents the number of questions used in each sub-section. PHE=Patient Health Engagement; brEASI=brief Epilepsy Anxiety Survey Instrument; SERDAS=Seizure Related Disability Assessment Scale; NDDI-E=Neurological Disorders Depression Inventory in Epilepsy.

**Supplementary Table 1. SERDAS, brEASI and NDDI-E scoring systems.** Total score is the sum of scores for all responses.

| **SERDAS and brEASI** | | **NDDI-E** | |
| --- | --- | --- | --- |
| **Possible response** | **Score per response** | **Possible response** | **Score per response** |
| Nearly every day | 3 | Always or often | 4 |
| More than half the days | 2 | Sometimes | 3 |
| Several days | 1 | Rarely | 2 |
| Not at all | 0 | Never | 1 |

**Supplementary Table 2. PHE scale scoring system.** Total score is the sum of scores for all responses.

|  | **Strongly agree** | **Agree** | **Neither agree nor disagree** | **Disagree** | **Strongly disagree** |
| --- | --- | --- | --- | --- | --- |
| I am very discouraged due to my condition | 5 | 4 | 3 | 2 | 1 |
| I feel adjusted to my condition | 1 | 2 | 3 | 4 | 5 |
| I feel anxious when I try to manage my condition | 5 | 4 | 3 | 2 | 1 |
| I am generally optimistic about my future and my health condition | 1 | 2 | 3 | 4 | 5 |
| I feel totally oppressed by my condition | 5 | 4 | 3 | 2 | 1 |
| I feel I have accepted my condition | 1 | 2 | 3 | 4 | 5 |
| I am upset when a new symptom arises | 5 | 4 | 3 | 2 | 1 |
| I can give sense to my life despite my condition | 1 | 2 | 3 | 4 | 5 |

## Appendix C: Initial vs follow-up responses

**Supplementary Figure 2. Changes in perceived forecast accuracy between initial and follow-up timepoints.** Percentage of In Practice group responses to the statement, “The forecast was accurate enough to be useful” at initial (top and middle bar) and follow-up (bottom bar) times. Note that the middle bar includes only responders who were In Practice users at both initial and follow-up timepoints.

**Supplementary Figure 3. Change in accuracy usage question amongst follow-up survey responders.** Follow-up group (N=18) changes in responses to the statement “The forecast was accurate enough to be useful.” during the initial survey period and at follow-up.

**Supplementary Figure 4.** **Forecast usage at initial and follow-up surveys.** Percentage of In Practice (orange) and In Theory (blue) users who responded to each tick box in response to a forecast usage question at both initial (coloured bars) and follow-up (empty bars) timepoints. In Practice users were asked “Have you ever changed your behaviour or activities because of the risk forecast and how (tick all that apply)?” and In Theory users were asked “In theory, if you had access to a seizure risk forecast, what would you use it for (tick all that apply)?” Note that only users who answered both surveys were included and users who switched groups (In Theory to In Practice, or vice versa) between initial and follow-up timepoints were excluded from this analysis.

**Supplementary Figure 5.** **Change in impact of risk condition over time**. In Practice and In Theory reported impact of **a:** high and **b:** low risk states at initial (coloured bars) and follow-up (empty bars) time points. Note that only users who answered both surveys were included and users who switched groups (In Theory to In Practice, or vice versa) between initial and follow-up timepoints were excluded from this analysis.

## Appendix D: Association of mood and adjustment scores with forecast experience

**Supplementary Figure 6. Correlation matrix to assess the relationship between answers to the *high risk* question, and mood and adjustment survey scores in the In Practice group.** *Indicates significant correlation (p<0.05), and the symbol (+ or –) next to the asterisk represents the direction of the correlation (positive or negative, respectively).

**Supplementary Figure 7. Correlation matrix to assess the relationship between answers to the *low risk* question, and mood and adjustment survey scores in the In Practice group.** *Indicates significant correlation (p<0.05), and the symbol (+ or –) next to the asterisk represents the direction of the correlation (positive or negative, respectively).

**Supplementary Figure 8.** **Boxplots of brEASI scores for In Practice and In Theory users.** Dashed line at 7 is the cut-off value commonly used in the literature to classify higher vs lower risk of anxiety disorders (11). No significant difference (via Mann-Whitney U test) was identified between In Practice and In Theory groups.

**Supplementary Figure 9.** **Boxplot of brEASI score differences (initial – followup score) for In Practice and In Theory users in the follow-up group.** Positive values indicate that the brEASI score dropped from initial to follow-up timepoints (i.e. reduction in anxiety likelihood over time). No significant difference (via Mann-Whitney U test) was identified between In Practice and In Theory groups, and neither group significantly differed from zero (via Wilcoxin signed-rank test) meaning that brEASI scores were not significantly different between timepoints.

**Supplementary Figure 10.** **Boxplots of NDDI-E scores for In Practice and In Theory users.** Dashed line at 16 is the cut-off value commonly used in the literature to classify higher vs lower risk of depression(12). No significant difference (via Mann-Whitney U test) was identified between In Practice and In Theory groups.

**Supplementary Figure 11. Boxplot of NDDI-E score differences (initial – followup score) for In Practice and In Theory users in the follow-up group.** Positive values indicate that the NDDI-E score dropped from initial to follow-up timepoints (i.e. reduction in depression likelihood over time). No significant difference (via Mann-Whitney U test) was identified between In Practice and In Theory groups, and neither group significantly differed from zero (via Wilcoxin signed-rank test) meaning that NDDI-E scores were not significantly different between timepoints.

## Appendix E: Factors influencing perceived accuracy

**Supplementary Figure 12. Forecast usage compared to perceived accuracy.** Correlation matrix to assess the relationship between answers to the forecasting usage question and perceived accuracy (strongly disagree to strongly agree on a scale from 0-4) in the In Practice group. *Indicates significant correlation (p<0.05), and the symbol (+ or –) next to the asterisk represents the direction of the correlation (positive or negative, respectively).

**Supplementary Figure 13. Response to high risk state compared to perceived accuracy.** Correlation matrix to assess the relationship between answers to the high risk question and perceived accuracy (strongly disagree to strongly agree on a scale from 0-4) in the In Practice group. *Indicates significant correlation (p<0.05), and the symbol (+ or –) next to the asterisk represents the direction of the correlation (positive or negative, respectively).

**Supplementary Figure 14. Response to low risk state compared to perceived accuracy.** Correlation matrix to assess the relationship between answers to the low risk question and perceived accuracy (strongly disagree to strongly agree on a scale from 0-4) in the In Practice group. *Indicates significant correlation (p<0.05), and the symbol (+ or –) next to the asterisk represents the direction of the correlation (positive or negative, respectively).

**Supplementary Figure 15. Relationship between seizure frequency and perceived accuracy amongst In Practice users.** Responder seizure frequency (per day, mean ± standard deviation) during the time that forecast was shown in comparison to their response to “The forecast was accurate enough to be useful.” Differences between seizure frequency and responses were assessed using Mann-Whitney U tests with a Bonferroni correction for multiple comparisons.

# Appendix F: Interview questions

**S1**

What are you looking forward to this week?

Back to the planner...do you write everything in there?

How did you manage with epilepsy?

You said you were really frustrated. How did finding research and all of this affect that feeling?

Why does that matter? Managing your time?

What, what made you frustrated about the whole process?

Is there anything else you write down?

Do you write all that stuff down because you can't put that in the app?

And how do you feel about that?

What does managing epilepsy mean to you?

What do you use the app for?

Great. And do you use it every day for anything else?

What kind of information?

Is there anything you put in the planner there isn't a spot for in the app relating to a seizure?

What do you want to feel when you're using our app?

What do you want to avoid feeling like?

if there was one thing you couldn't live without to manage your epilepsy, what would that be? What is one thing?

**S2**

What does managing epilepsy mean to you? Like, what does that actually mean to you specifically?

Interesting. What are the after effects?

Is that the same for each of each type of event?

So can you talk to me about auras a little bit more? I'd like to know how long an aura is, is it days? Is it hours?

Is there a pattern in any of this? Like, do you know, it's just, is it very random or do you know?

So you started videoing it. Why?

When did you start videotaping? Have you been doing that?

Why, why do you think that?

Is that something you've found you couldn't find when you first had seizures?

When did you first start experiencing your rare condition?

How did that make you feel?

That's amazing. Do you do it every day or every week? I am curious about how you document this? What does that look like?

What sort of information do you give about a seizure?

How are you documenting this?

Do you just remember it?

Right. How does that fit into this process?

So does the time, the instance of when you put this information in, has it changed now that you have the app? Do you put it in straight away or you just weekly?

I'm curious if there's anything that you would want to record through the app that you still do manually.

So you'd like to see all the wearable data in the app and just have it all in one place?

I guess I'm still curious about this wearable data. Yeah, you'd like the, like would you like, like summaries of, of the wearable data showing like how we think it might relate to epilepsy? Or, or would you rather just like here's the, the raw information or something else?

So you said the system's excellent. You like the app system. What do you like about it?

So you're saying you'd like the auras to be automatically picked up?

Why would you like that? How would that be helpful to you?

So we talk about risk a lot. And I'm wondering what does that mean to you? What is risky? What is risk to you in terms of this?

Why do you look at the forecast?

So let's go back then. Why do you look at it every day?

So when do you look at it then?

Do you look at it to plan anything?

When's the last time you did use it? The forecasting feature?

Why was that?

Have you ever had a seizure? And the forecast was wrong? It said it was going to be a low risk, but you had one?

When you first used the forecasting feature, was it what you expected?

Okay. Why do you like to see the data?

Why do you distrust?

What do you want to feel when you're using our app?

And what do you think the app is trying to achieve?

What do you want to avoid feeling like?

Do you feel that high risk makes you discouraged and low risk makes you confident?

How do you feel being in the middle? Do you feel discouraged?

if there was one thing you couldn't live without to manage your epilepsy, what would that be? What is one thing?

**S3**

What is one thing that you were looking forward to this week?

So are you close with your family then?

Why are you looking forward to that?

What does that mean to you? Managing epilepsy?

Why are you lucky that he's around all the time?

So can I make the assumption that he's a big part of you helping manage epilepsy?

Was the first specialist, a specialist of epilepsy?

How do you manage something unpredictable?

How often do you see him?

And what's not going good for you? What does that look like?

But were you not afraid because of your seizures to go away for that long?

So how do you work around that? …how do you work around not knowing when you're going to have a seizure or having the seizure in the moment?

Like are there certain strategies that you have?

Do you sort of gauge what activities you'll do based on I guess, how often you're having seizures?

Was that because you had a lot of seizures, or do you think you wouldn't have done that anyway?

How do you record this information?

Are there weeks where you have good weeks?

Why do you have your own spreadsheet?

Is there a reason you still keep it in the spreadsheet?

Is it because you feel more comfortable with spreadsheets?

Why is it easier? What information do you put in your spreadsheet that's not in the app?

And why do you like to keep that information?

Why is it wonderful?

Why do you think it's important to record all this information to be helpful?

I'm gonna ask why again? Cause I don't want to assume, why is that so important to you to make sure that it's making a difference?

So can I make an assumption that you don't feel the need to have to put input meds somewhere?

Can I ask why not? Why aren't you using the app to do that?

So is that an improvement on the previous therapy regime that you were on?

If after the two weeks at a thousand milligrams, for example, you halved your numbers, number of seizures, would you still feel the need to immediately get in touch with your specialist or would that be a different situation?

What would success look like for you after two weeks?

I'd like to know what does that mean to you? What is risky?

Why?

Has he ever had to pick you up or like early or have you had to?

How would you find the education of the general public on epilepsy from one to five?

Why do you think someone who didn't know you as well would've treated you like that?

How does that make you feel?

Do you think that makes a difference?

Do you think your GP does that?

Do you feel like you can go to your GP with some of these sort of questions and concerns? Do you think your GP can help you with your epilepsy?

And is it something that they think they'll be able to help you with in the future?

How did you become aware that that is a thing that you'll have to go through?

What changed for your part of medication? What changed?

Did you start doing your own research?

Can I ask you about the forecasting feature? You mentioned that in your survey results. Can you tell me last time you looked at it?

Why did you look at it and how do you feel about it being wrong?

How does that make you feel?

Would that be helpful knowing how it works?

Since we're talking about the forecasting feature, has it ever been useful?

Is that something that went through your head when you first found out?

And that goes back to, again, one of these silly questions, but I'm assuming that goes back to wanting to take control of what's happening to you?

Why do you find the insights helpful?

Why is that good information?

How do you want to feel when using the app?

Why informed?

Is there a feeling that you, not just with our app, but like any sort of piece of technology that you want to avoid feeling a certain way when you're using it?

If there was one thing you couldn’t live without to help manage your epilepsy, what is that?

**S4**

So you got injured at work? Is that related to your epilepsy?

Is that something that happens?

Had you been having seizures before that?

When I say managing epilepsy, what does that mean to you?

Is that what managing is to you? Is that just being prepared to have that seizure? What does that look like? Getting prepared for a seizure?

And how do you feel about your neurologist?

Why do you hate being in the dark about this stuff?

When you say it helps somewhat, what does it help you with?

Do you use technology at all to help you? Like do you, like, do you use apps or anything else to help manage and, and help you with your epilepsy?

Why do you feel like it would be nice to get everyone in the room? What are you hoping will happen?

Why?

Is that a GP? Your doctor?

I guess one of my biggest questions I have in my head right now is what if, if you were to get them all in a room, what would be your ideal outcome? Like what, what are you hoping will happen? Is there something you think will happen outside of obviously the sharing of information and, and caring for you better if you get better care? What, what do you think that will help with? Like specifically?

How do you manage it day-to-day?

What does your day-to-day look like? Managing epilepsy?

How are you recording those seizures? Are you doing it through the app or are you just putting it in the email?

Why don't you want to go to a barbecue?

Why did you join?

Do you use the app every day?

And so what do you use the app for?

Why do you want to do all of this?

How do you manage that after you've had a seizure?

What are you curious about? And my second question is, do you look at the forecaster every day?

Why?

Did it match your expectations?

And that's what's motivating you to keep putting data in?

Do you check it at the same time every day?

Oh really?

Have you noticed a decrease in seizures on the app with any medications you're taking or, or other sort of management strategies? Have you seen any patterns?

Is there anything else you want to share around how you think the forecasting feature can be improved or the app in general?

Why is that? Why do you want it on that?

What does risk actually mean to you?

What do you want to feel when you're using the app?

Why secure?

What do you want to avoid feeling when using the app?

Do you feel scared when using the app now?

If there was one thing you couldn't live without to manage epilepsy, what would that be?

And just to confirm safe to you is what? Alone?

**S5**

What is one thing you're looking forward to this week or weekend I should say?

Where is it at now?

What does safe mean?

Why is it very different? Why is it so different being out?

Have you had a seizure in public before?

How do you get one of those? What does it have on it? What does it say?

Why do you not want to have a seizure out in public outside of the safety thing? Like what happens after?

What does managing epilepsy mean to you?

What does happen before and after? What does that look like for you?

Why is it so embarrassing?

How does that affect managing your epilepsy or did it affect managing your epilepsy?

Was that what caused that epilepsy you think?

So are you on medications now?

How did you figure that out about the medication and do you use the app for medication management?

And then why did you change neurologists?

What do you like about it and how do you think it can be improved?

How do you manage your medication?

What does managing epilpesy look like for you? What does support look like for you?

I wanted to kind of start off with, again, we talk a lot about risk forecasting, right? That's actually what we, what we ask in the survey. That's what we say, you know, when we talk about the forecasting features, helping you forecast risk, what, what does risk mean to you?

Yeah, that's fair. How do you feel about the term risk?

How did you figure that out?

And so, and because of that, because of that mindset that you have now, you are able to be more aware in the moment of what's happening?

And are you, when, when, when are you recording these? Are you doing it after?

Oh, does that motivate you?

How can we make the algorithm more accurate and what's the data that we can be helping people put in?

So the first time you used the forecasting feature on a scale of one to five, one being not at all, five being totally, did it match your expectations?

We talked about trust earlier and trusting something like how, what, what would it look like? Is there an example of something you trust a lot with your data that you would never save the data yourself?

When's the last time before today that you looked at the forecasting feature?

Does the algorithm say it's out of cycle?

How does that make you feel?

And what does that mean to you? What does supported mean?

What does support look like that your neurologist isn't giving you?

When you are using the the app, what do you wanna avoid feeling

If there was one thing you couldn't live without to help you manage epilepsy, what would that be?

What do you use the watch for when you do use it?

**S6**

What's one thing that you are looking forward to this week?

What do you mean by positive impact?

Can you tell me that story?

18 in like a day?

And what's happened is just in the last, did you say June or October?

Is that the way you've managed your epilepsy would have to have changed in the last six months since June? So what was it like before and how has it changed I suppose?

Is that nine months?

When I say managing epilepsy, what does that mean?

Just because you've mentioned that you've tried various medications, are you using the medication manager part of the app as well or?

How and when did you start using the app, and why?

Did you start trying out the app because of the forecasting feature?

How much do you push back on the directions you've been getting given from?

How do you find these other possible ways to manage epilepsy?

Can you, can you talk about that? Like what happened for that to be a bad experience with the specialist?

Supposedly you are speaking about your neurologist when you say professionals?

Was the GP advocating for you?

Has it been something that you're referring to on a daily or how often are you checking?

When is the last time you checked it?

What does risk actually mean to you? What is risky for your particular situation?

What are your support networks like?

Are you a part of any online communities?

When you are using the app, how do you want to feel?

Why empowered?

If there was one thing you couldn't live without to manage your epilepsy, what would that be?

**S7**

What's one thing that you're looking forward to this week?

When I say managing epilepsy, what, what does that mean to you?

So what does that look like for you?

And what was it like beforehand?

How did you, like, how did you work with that or manage that when you did have to?

Can you walk me through what would happen if you did have a seizure?

Do you have aura?

What did the dog do?

Can you, can you walk me through that? Have they been a part of your, I guess, supporting with managing epilepsy?

How have they been a part of supporting you with managing?

Why is that something you didn't wanna hear from your perspective?

Why else is it important to you?

Why didn't you think it was at that time?

Were you not afraid?

How did you get to now? How'd you, how'd you get there? That's a big change.

Were you managing medication through anything or anyone?

Do you have to have reminders to take it or do you just, are you good at just taking it when you need to?

How would your family know if you hadn't taken it?

Would you write if something abnormal happened?

How did to become aware that seizures occur in cycles?

Is this the, the gap between them or how many days?

Can you tell me why?

And so were you still inputting?

Did you put all the data in that you had about your seizures before the forecasting feature was available?

Do you still look at it?

Why do you put the information in every day? Why do you look at the risk every day?

What will you think? What's who mean?

What happens though if it is high risk and nothing happens, then what do you think?

What is risky mean to you?

Do you do anything if you see that it's a high risk day, any strategies that you use to manage those risks?

Like do you still manage or do you still have strategies like that? Were there any other strategies that you used to manage the risk of having a seizure? What does that look like now? Let me rephrase that. When you're looking at the app now, in terms of risk, does it change your behaviour at all?

And why not?

And why is that?

Do you wonder where it's come from?

Why do you say that?

Do you find the feature useful today?

Why would you like to stop using the app?

How would it be useful? What would you do with that information?

What do you want to feel when using the app?

Why do you say that?

What do you want to avoid feeling when you're using the app?

**S8**

What's one thing you are looking forward to this week?

Why are you lucky?

And your family, do you get to see them a lot?

What does managing epilepsy mean to you?

So you said when you were younger? Can you tell me about that journey?

How long have you been living with epilepsy?

So has managing epilepsy changed?

So what does it look like now?

Still happens?

Oh, you work?

So what do you do for work?

Do you find that working environment a little bit more understanding or accepting around epilepsy?

And how often do you typically have seizures?

Have you ever had a seizure at work?

How did you manage that?

I'm assuming that's a person that you trusted?

What happens after? How do you feel? Does it take time to feel better?

Do you ever record anything?

Do you have any sort of aura/sign beforehand?

So they're very unpredictable it sounds like?

So you record the seizure on the app?

Can you tell me a little bit about that? What does that mean interesting to you?

Let's talk about that, actually, why do you wish you could figure it out?

Why?

Are you, you seeing a, a neurologist regularly or a specialist?

What's what's led you to not wanting to talk about your epilepsy? Have you had experiences in the past?

Psychogenic seizures?

How long was that?

And this was after you had given birth to your first son?

Did you have the other children during that time?

Do you know what year that was? Was that recently?

Before you had the app, did you record them in any way?

Do you find that useful recording then?

With the app, we talk about forecasting risk. I want to know, what does risk mean to you? What is risky?

And I guess in relation to having a seizure, what is risk?

Why?

Is that the biggest thing you feel that's risky? Is that you harming other people?

How do you feel about the term risk?

You don't look at the forecasting feature?

So when is the last time you checked it?

Do you know if it was a month ago, a week ago, that you had you your last event?

Is that, and that's because you were putting information in about the last event you had?

Why is that?

I was just wondering, is anything that you wish you could do?

Are you finding that it's preventing you from doing things at the moment?

What, what would getting it right look like?

If we could tell you say for example, that next Tuesday is not going to be a good day reliably, would you find that more useful than what you've got at the moment?

Have you got a reliable GP or someone you see regularly?

Did you have any of these conversations with them?

Is that something that you think the app could help you with?

When you say you don't see the point of talking about it, I'm assuming you mean going back to what we spoke about earlier about just trying to say positive and talking? That it's not really helping talking about it?

You're going to have it on this day, right?

How did you get onto the forecasting feature? How did you find out about it?

What exactly caught your interest when you saw it?

Why was that important to you? Why were you interested in keeping a diary?

When you used the forecasting feature, how long have you been using it?

From when you first started using the app, how did the forecasting feature match your expectations from a score to 1-5?

Why are you hopeful?

Do you use other parts of the app? Like the medication manager or any of that?

Any other parts that you find helpful?

The diary function?

How is that going? Have you found that to be easy to use?

Anything you'd like to change?

And do you find it useful to sometimes look back where the things are changing over time?

Are there things that you would like to see the app do?

If the app told you stuff about epilepsy, would that be interesting to you, or would you rather avoid it?

Is there anything that would make it easier though?

Is there any information that you think, I wish I could input that information and I can't right now?

Regarding your events?

Is there anything overall not just the forecasting feature or logging events - that you feel would be helpful for the app to do for you?

What parts are you finding confusing?

Would that be more helpful? Or would you avoid doing something like that?

And is that because of a data privacy concern or you don't really want to record yourself when you're in that state?

How would you feel about telling?

Do you use a wearable at all?

Which one do you have?

What do you want to feel when you're using the app?

And why?

And why do you want to solve the equation?

What do you want to avoid feeling when you're using the app?

Because then what?

That whole ignorance is bliss?

I'm curious what do your dogs give you?

Do they know when you have a seizure?

**S9**

What is one thing that you look forward to this week?

Why?

It's so funny how, just the little things, isn't it?

Can you explain that?

So what happened? Did the seizure happen?

How do you manage that? We're talking about managing epilepsy, but how do you manage something you don't know is going to happen?

What is it like doing it? What is, what is your definition of managing epilepsy? What does that mean to you?

Can you walk me through what does it look like managing your epilepsy, including the medication? Like what are the things that you have to do to manage it?

So is that the reason why, so you can discuss with your neurologist?

Were you able to identify that through logging this information?

Can I ask what exactly you, after you have a seizure, what's the information that you log is there anything that you wish you could log?

How is he logging this data to give back to you?

So outside of the app, is there anything else that you do to, to track the seizures or to manage it?

Just curious with what you're saying about at work, does it happen often at work?

And then, but you said the one thing that is in common is that you feel really tired, like you need to sleep afterwards?

What did that look like? What did support look like when you first found out? What did managing look like? Where, where did you start from?

What does that mean you guess? And not as well as you like it to?

How does that make you feel?

So is that not happening anymore?

You're able to get to the bed?

So it hasn't reduced it though? It hasn't reduced the amount, just kind of what happens afterwards?

And how long have you been taking medication?

Do you have any other support networks outside of your family and neurologist?

Is that why you do it?

What was the journey like?

Why was it weird that it was epilepsy?

How did you find out about the app?

And how'd you find out about the research program?

Have you had a wearable before? Or is this a new thing?

And how are you finding it?

How so?

Is that data helping you put together the reports you give to your neurologist?

How do you get that data? Does it go directly into the app for you then?

I actually want to understand, what does that mean to you? What is risky to you? What is the term risk?

And in general, what does that mean? What is risky for you?

Do you look at the forecasting feature as forecasting risk?

Tell me about that. What is the dangerous situation? What does that look like?

So why do you look at it?

Why is that?

Do you ever think it would be helpful?

Are you putting the data in every day?

Can you remember that? Can you go back to that?

So when it did finally work was on a scale of one to five, one being not at all what you expected, five being what you expected, did it match your expectations of what you thought a forecasting feature would be?

What about it made it a five?

When is the last time you looked at it?

And why was that?

Are you curious because of your medication trial and seeing how that affects you? Why are you curious?

Are you logging all of your meds every day, or do you find that you just use it as a reminder?

Would you like to be able to log it in in a quicker way or it, would it be useful to be able to, you know, say exactly how your medications are being taken?

If you were able to record more accurately based off your schedule, would that be beneficial?

Is that what the reporting is like with the neurologist?

What do you want to feel when using the app?

Why satisfied? What does that mean?

And does that go back to our conversation about when you were talking about the usefulness being related understanding why more?

What do you want to avoid feeling when using the app?

What do you mean by tired?

If there was one thing you couldn't live without to manage epilepsy, what would that be?

Why?

# Appendix G: Forecast performance comparison between responders and non-responders

Supplementary Table 3 outlines the performance of the prospective seizure risk forecast for all app users, initial survey responders and follow-up survey responders. Hourly and daily forecasts performed significantly better than chance for 63% and 23% of app users with average ROC-AUC scores of 0.71 and 0.69, respectively. Initial survey responders did not show any significant difference in their hourly or daily ROC-AUC scores compared to both non-responders and follow-up survey responders (Mann-Whitney U test, p>0.05).

**Supplementary Table 3.** Performance metrics of hourly and daily forecast for users with more than five events who performed better than chance (Improvement over Chance (IoC) users). Results are reported during the evaluation period (approximately nine months) for three user groups: all app users, initial survey responders and follow-up survey responders. Values are given as mean ± standard deviation. Note that daily accuracy scores were only evaluated for users who had at least one significant multiday cycle at least 50% of the time during the evaluation period.

| **User group** | **Total number of users** | **Number of users with >5 seizures (hourly:daily)** | **Number of IoC users (hourly:daily)** | **ROC-AUC score** | |
| --- | --- | --- | --- | --- | --- |
|  |  |  |  | **Hourly** | **Daily** |
| All app users | 1053 | 504:398 | 318 (63%):  92 (23%) | 0.711 ± 0.113 | 0.693 ± 0.104 |
| Initial survey responders | 111 | 48:41 | 37 (77%):  8 (20%) | 0.687 ± 0.062 | 0.611 ± 0.036 |
| Follow-up survey responders | 41 | 19:17 | 16 (84%):  3 (18%) | 0.695 ± 0.074 | 0.642 ± 0.031 |

# References

1.	Karoly PJ, Freestone DR, Boston R, Grayden DB, Himes D, Leyde K, et al. Interictal spikes and epileptic seizures: their relationship and underlying rhythmicity. Brain. 2016 Apr;139(4):1066–78.

2.	Karoly PJ, Ung H, Grayden DB, Kuhlmann L, Leyde K, Cook MJ, et al. The circadian profile of epilepsy improves seizure forecasting. Brain. 2017 Aug 1;140(8):2169–82.

3.	Karoly PJ, Goldenholz DM, Freestone DR, Moss RE, Grayden DB, Theodore WH, et al. Circadian and circaseptan rhythms in human epilepsy: a retrospective cohort study. The Lancet Neurology. 2018 Nov;17(11):977–85.

4.	Karoly PJ, Cook MJ, Maturana M, Nurse ES, Payne D, Brinkmann BH, et al. Forecasting cycles of seizure likelihood. Epilepsia. 2020;61(4):776–86.

5.	Leguia MG, Andrzejak RG, Rummel C, Fan JM, Mirro EA, Tcheng TK, et al. Seizure Cycles in Focal Epilepsy. JAMA Neurol [Internet]. 2021 Feb 8 [cited 2021 Feb 9]; Available from: https://jamanetwork.com/journals/jamaneurology/fullarticle/2775979

6.	Karoly PJ, Stirling RE, Freestone DR, Nurse ES, Maturana MI, Halliday AJ, et al. Multiday cycles of heart rate are associated with seizure likelihood: An observational cohort study. EBioMedicine. 2021;72:103619.

7.	Stirling RE, Grayden DB, D’Souza W, Cook MJ, Nurse E, Freestone DR, et al. Forecasting seizure likelihood with wearable technology. Frontiers in Neurology [Internet]. 2021; Available from: http://medrxiv.org/content/early/2021/05/26/2021.05.20.21257495.abstract

8.	Xiong W, Stirling RE, Payne DE, Nurse ES, Kameneva T, Cook MJ, et al. Forecasting Seizure Likelihood from Cycles of Self-Reported Events and Heart Rate: A Prospective Pilot Study. Available at SSRN 4194643. 2022;

9.	Torrence C, Compo GP. A practical guide to wavelet analysis. Bulletin of the American Meteorological society. 1998;79(1):61–78.

10.	Maturana MI, Meisel C, Dell K, Karoly PJ, D’Souza W, Grayden DB, et al. Critical slowing down as a biomarker for seizure susceptibility. Nat Commun. 2020 Dec;11(1):2172.

11.	Scott AJ, Sharpe L, Thayer Z, Miller LA, Hunt C, MacCann C, et al. Design and validation of two measures to detect anxiety disorders in epilepsy: The Epilepsy Anxiety Survey Instrument and its brief counterpart. Epilepsia. 2019;60(10):2068–77.

12.	Friedman DE, Kung DH, Laowattana S, Kass JS, Hrachovy RA, Levin HS. Identifying depression in epilepsy in a busy clinical setting is enhanced with systematic screening. Seizure. 2009;18(6):429–33.
